# Brief composite mobility index predicts post-stroke fallers after hospital discharge

**DOI:** 10.3389/fresc.2022.979824

**Published:** 2022-09-23

**Authors:** Prudence Plummer, Jody A. Feld, Vicki S. Mercer, Pengsheng Ni

**Affiliations:** ^1^ Department of Physical Therapy, Cognitive-Motor Behavior Laboratory, MGH Institute of Health Professions, Boston, MA, United States; ^2^ Department of Orthopaedic Surgery, Duke University, Durham, NC, United States; ^3^ Department of Allied Health Sciences, University of North Carolina at Chapel Hill, Chapel Hill, NC, United States; ^4^ School of Public Health, Biostatistics and Epidemiology Data Analytic Center, Boston University, Boston, MA, United States

**Keywords:** stroke, falls, gait, balance, inpatient rehabilitation

## Abstract

**Introduction:**

Community-dwelling, ambulatory stroke survivors fall at very high rates in the first 3–6 months. Current inpatient clinical assessments for fall risk have inadequate predictive accuracy. We found that a pre-discharge obstacle-crossing test has excellent specificity (83%) but lacks acceptable sensitivity (67%) for identifying would-be fallers and non-fallers post discharge.

**Hypothesis:**

We assessed the hypothesis that combining the obstacle-crossing test with other highly discriminatory fall risk factors would compensate for the obstacle test’s fair sensitivity and yield an instrument with superior prediction accuracy.

**Methods:**

45 ambulatory stroke survivors (60 ± 11 years old, 15 ± 11 days post stroke) being discharged home completed a battery of physical performance-based and self-reported measures 1–5 days prior to discharge. After discharge, participants were prospectively followed and classified as fallers (≥1 fall) or non-fallers at 3 months. Pre-discharge measures with the largest effect sizes for differentiating fallers and non-fallers were combined into a composite index. Several variations of the composite index were examined to optimize accuracy.

**Results:**

A 4-item discharge composite index significantly predicted fall status at 3-months. The goodness of fit of the regression model was significantly better than the obstacle-crossing test alone, *χ*^2^(1) = 6.036, *p* = 0.014. Furthermore, whereas the obstacle-crossing test had acceptable overall accuracy (AUC 0.78, 95% CI, 0.60–0.90), the composite index had excellent accuracy (AUC 0.85, 95% CI, 0.74–0.96). Combining the obstacle-crossing test with only the step test produced a model of equivalent accuracy (AUC 0.85, 95% CI, 0.73–0.96) and with better symmetry between sensitivity and specificity (0.71, 0.83) than the 4-item composite index (0.86, 0.67). This 2-item index was validated in an independent sample of *n* = 30 and with bootstrapping 1,000 samples from the pooled cohorts. The 4-item index was internally validated with bootstrapping 1,000 samples from the derivation cohort plus *n* = 9 additional participants.

**Conclusion:**

This study provides convincing proof-of-concept that strategic aggregation of performance-based and self-reported mobility measures, including a novel and demanding obstacle-crossing test, can predict post-discharge fallers with excellent accuracy. Further instrument development is warranted to construct a brief aggregate tool that will be pragmatic for inpatient use and improve identification of future post-stroke fallers before the first fall.

## Introduction

The incidence of falls in ambulatory stroke survivors who are discharged home is high (1–4), estimated to range from 35% (2) to 73% (3) in the first 6 months. However, as many as 58% of the people who will fall, fall in the first month after discharge (5). These high fall rates suggest that rehabilitation may not be adequately targeting person-specific risk factors for successful fall prevention. Indeed, current fall-risk assessment tools are inadequate for predicting future fallers at hospital discharge. For example, the Berg Balance Scale (BBS), which is considered a reference standard for assessing balance in stroke, has only fair ability to predict future fallers among patients with stroke receiving inpatient rehabilitation (sensitivity 63%, specificity 65%, using cut off score <45) (2). One possible explanation for its weak discrimination properties is that the BBS comprises relatively low-demanding postural control tasks and does not include any walking tasks. This is an important limitation considering that a large proportion of post-stroke falls occur during walking (6–8).

Walking-related falls are often due to tripping (8, 9) or falling over obstacles (3). Indeed, it is known that subacute stroke survivors who are fallers are more likely to fail an obstacle-crossing task than non-fallers (10), yet very few clinical assessments for evaluating fall risk include obstructed walking. The Functional Gait Assessment (FGA) includes 10 gait-related tasks of varying difficulty, including stepping over an obstacle (a shoebox), but its ability to predict post-stroke fallers during inpatient rehabilitation has not been tested. We recently demonstrated that subacute stroke survivors who failed an obstacle-crossing test at hospital discharge were 10 times more likely to fall in the first 3 months after going home than those who passed the obstacle-crossing test (11). Although the obstacle-crossing test had excellent specificity (83%), it was lacking acceptable sensitivity (67%). The area under the receiver operating characteristic (ROC) curve (AUC) was 0.75 (95% CI, 0.60–0.90).

Forty-three percent of the fallers who were misclassified by the obstacle test as a non-faller (i.e., false negatives) had used a walker to perform the test. The bilateral upper extremity support provided by the walker diminishes the demands on dynamic postural control, which most likely contributed to their successful performance on the obstacle-crossing test despite considerable balance impairment. Further, non-adherence to assistive device use in the home following rehabilitation is common (12) and may undermine the accuracy of fall-risk assessment in which assistive devices are used. Thus, to optimize fall prediction accuracy, clinical assessments may need to incorporate physical performance tests of dynamic postural control with and without assistive devices.

The purpose of this study was to test the hypothesis that combining the obstacle-crossing test with other clinical assessments demonstrating strong faller/non-faller discriminatory ability, including at least one measure of dynamic postural control that was performed without an assistive device, would compensate for the obstacle-crossing test’s limitations in sensitivity and yield a composite instrument with superior prediction accuracy. This study describes a secondary analysis of data and is intended to demonstrate proof-of-concept to guide further instrument development.

## Materials and methods

### Participant selection

The participants were 45 individuals with stroke who participated in the initial exploratory study of the utility of the novel obstacle-crossing test (11). To be included, participants had to be 35–85 years old, admitted to the hospital for a diagnosis of stroke, previously residing independently in the community, discharge disposition to return home, able to follow a 3-step verbal command in English, and able to walk with or without an assistive device and/or ankle-foot orthosis (AFO) with no more than light touch assistance for balance. Exclusion criteria were previous stroke with residual physical or cognitive-communication impairment, dementia, pre-stroke history of falls (>1 fall in previous 12 months) or pre-stroke assistive device for ambulation, any pre-stroke comorbidity that limited gait or physical activity including other neurological diagnoses and peripheral neuropathy. We also excluded individuals with cerebellar stroke because it is relatively uncommon, accounting for only 3.4% of ischemic strokes (13), and can present quite differently than the more common cerebral stroke syndromes (14). Ambulatory stroke survivors being discharged home from acute care or acute inpatient rehabilitation at the University of North Carolina Hospital system or Duke University Hospital were referred by the staff physical therapists to the researchers for eligibility screening.

Between August 2017 and January 2019, 217 individuals were referred by clinical staff for eligibility screening, of which 56 who were eligible consented to participate. Nine were withdrawn following consent due to inability to complete testing before discharge (*n* = 3), no longer wishing to participate (*n* = 2), or failing to meet inclusion criteria (*n* = 4). An additional two participants were lost to follow up, which left 45 participants with completed discharge assessment and 3-month follow up for inclusion in the analyses.

The characteristics of the participants are summarized in Table 1. Comparisons between fallers and non-fallers in clinical measures of functional and self-rated performance are presented in Table 2. All participants had some neurological symptoms. The discharge modified Rankin Scale (mRS) scores ranged from 1 (no significant disability despite symptoms) to 4 (moderately severe disability). The median discharge mRS for non-fallers was 2 (range 1–4) and the median discharge mRS for fallers was 3 (range 1–4; *p* = 0.004). The data in Table 2 illustrate that although fallers were significantly more impaired than non-fallers, on average, non-fallers also demonstrated clinically important limitations in mobility [e.g., average gait speed <0.80 m/s (15)].

**Table 1:** Participant characteristics (*n* = 45). Values are number (%) or median (IQR) as indicated.

	Median/*n* (IQR/%)
Age (years), median (IQR)	61 (53–67)
Sex, male, *n* (%)	27 (60%)
Race, *n* (%)	
African American/Black	20 (45%)
Asian	2 (4%)
White	22 (49%)
Other	1 (2%)
Education (years), median (IQR)	14 (12–16)
Total hospital length of stay (days), median (IQR)	16 (8–26)
Type of stroke, ischemic, *n*, (%)	35 (78%)
Left-side hemiplegia, *n*, (%)	23 (51%)
Cumulative Illness Rating Scale for Geriatrics (score 0–56), median (IQR)	12 (8–15)
PHQ-9 (score 0–27), median (IQR)	6 (2–9)

**Table 2:** Comparisons between fallers and non-fallers on biologic (age, sex) and modifiable variables collected at hospital discharge unless otherwise indicated. Values are n (%) or mean/median (SD/IQR). Between-group effect sizes for significant differences only (α = 0.05) are presented as Cohen's d for normally distributed continuous variables and Cliff's δ for skewed continuous variables. Odds ratios (OR) are the effect sizes for the categorical variables.

Variable	Faller (*n* = 21)	Non-Faller (*n* = 24)	*p* ^a^	Effect size^b^
Age (years)	60.8 (12.0)	59.3 (12.0)	0.659	* *
Sex, male, *n* (%)	13 (62%)	14 (58%)	0.807	* *
MoCA (score out of 30)	26 (24–27)	24 (20–26)	0.045	*δ* = −0.35
Aphasia Quotient (score %)	100 (99–100)	100 (98–100)	0.239	* *
Unilateral neglect, *n* (%)	0 (0%)	1 (4%)	>0.999	* *
Visual Acuity, *n* (%)			0.061	* *
Good (≤20/25)	14 (67%)	22 (92%)		* *
Moderately impaired (20/30–20/80)	7 (33%)	2 (8%)		* *
Poor (≥20/100)	0	0		* *
Assistive device type, *n* (%)				
None	4 (19%)	13 (54%)	0.028	OR 9.8
Single-point cane	6 (29%)	2 (8.5%)	0.022	OR 11.4
Quad cane	7 (33%)	2 (8.5%)	0.014	OR 1.9
Walker	4 (19%)	7 (29%)	0.466	
AFO/ankle brace use (*n*, %)	8 (38%)	5 (21%)	0.202	* *
ABC Scale (score out of 100)	57.8 (18.8)	71.2 (23.4)	0.041	*d *= 0.63
Walk-12 (score out of 100)	72.4 (19.7)	43.3 (25.3)	<0.001	*d *= 1.28
Obstacle-crossing test, fail, *n* (%)	14 (67%)	4 (17%)	0.001	OR 10.0
5-m gait speed (m/s)	0.47 (0.24)	0.74 (0.36)	0.007	*d *= 0.86
Dual-task gait speed (m/s)	0.25 (0.15–0.44)	0.62 (0.31–0.91)	0.003	*δ *=* *−0.51
2 MWT distance (m)	64.2 (38.9)	102.8 (50.1)	0.009	*d *= 0.86
5× sit to stand (reps/s)	0.17 (0.10)	0.24 (0.12)	0.034	*d *= 0.66
Step Test (reps)				
Paretic limb	0 (0–5)	7 (5–11)	<0.001	*δ* = −0.62
Non-Paretic limb	5 (0–7)	8 (6–13)	0.002	*δ* = −0.51

Abbreviations: 2 MWT, 2-Minute Walk Test; ABC, Activities-specific Balance Confidence; AFO, ankle-foot orthosis; OR, odds ratio; Walk-12, Walking Impact Scale.

^a^ *p* values are differences between fallers and non-fallers determined using independent *t*-test, Mann-Whitney *U* test, chi-square, or logistic regression.

^b^ Cohen’s d: 0.2, 0.5 and 0.8 correspond to small, medium, and large effects; Cliff’s *δ* ranges from −1 to +1 where values closer to ±1 are larger effect sizes.

### Clinical and demographic measures at discharge

Clinical and demographic descriptive measures were age, sex, type of stroke (i.e., ischemic or hemorrhagic), total length of stay in hospital, depression (9-item Patient Health Questionnaire, PHQ-9), multi-morbidity using the Cumulative Illness Rating Scale for Geriatrics, visual acuity using a Snellen eye chart, cognitive screening *via* the Montreal Cognitive Assessment, unilateral spatial neglect screening *via* the Star Cancellation test, and the aphasia quotient of the Western Aphasia Battery (bedside version). All assessments were conducted by the research staff prior to discharge. Demographic and stroke variables were not considered as predictors during index model derivation because we wished to focus only on modifiable variables that would help direct rehabilitation.

### Assessment of physical function at discharge

As close as possible to the day of hospital discharge, participants underwent a brief assessment battery administered by the researchers comprising physical and self-reported measures of mobility function and balance. These clinical tests were originally chosen to provide comprehensive descriptive data in the primary study of the utility of an obstacle-crossing test as a discharge fall-risk assessment (11), and because they are commonly used in clinical practice and easy to obtain in the inpatient setting. In addition to the obstacle-crossing test, we administered the step test, a 5-m walk test (5 mWT) for gait speed, a 2-minute walk test (2 MWT) for walking endurance, dual-task gait speed, the 5-times sit-to-stand test (5xSTS) for lower extremity strength, the 12-item Walking Impact Scale (Walk-12) for self-rated walking disability, and the Activities-specific Balance Confidence scale (ABC) for balance self-efficacy.

The obstacle-crossing test required participants to walk towards and step over an obstacle and continue walking. The obstacle was constructed from stacked blocks on either side with a horizontal bar placed across the top. Height was customized to 10% of leg length (mean ± SD height: 8.8 ± 0.5 cm). We chose 10% of leg length [approx. 8 cm (16)] because it approximates curb height (16). However, rather than use a fixed height for all participants (e.g., 8 cm), we chose to normalize the height to leg length to try to equalize the biomechanical demands of the task across participants. The horizontal bar was 1.3 cm deep, and 91.4 cm wide. The obstacle was placed 5.5 m from gait initiation in a 7.5 m walkway. Four trials were attempted. Each trial was scored on 5-point scale, where 0 = clears obstacle without stopping (pass), 1 = clears obstacle after stopping and/or experiences some unsteadiness (pass), 2 = lightly contacts obstacle but the obstacle is not displaced (fail), 3 = contacts and displaces the obstacle (fail), and 4 = requires assistance to step over the obstacle or recover balance regardless of clearance success (fail). Only three of the 45 participants were unable to complete 4 trials, but all participants completed at least 2 trials. The worst score from all attempts was recorded as the overall score. The rationale for taking the worst score, rather than the best, was because a failed attempt on at least one trial was considered to indicate high risk for unsuccessful obstacle clearance in real life.

The step test involved the participant attempting to place his/her foot continuously on and off a 7.5 cm block as many times as possible (without assistive devices or support) in 15 s (17). The step test has very good reliability in stroke (test-retest reliability, ICC > 0.88) (17). The number of completed steps in 15 s for each lower limb were recorded. Participants who were unable to stand unsupported or who required assistance to place the foot on the step received a score of 0. A cut-off score of <7 for either limb in adults with stroke assessed within 14 days of being discharged home from hospital has been associated with recurrent falls in the first 6 months after discharge from inpatient rehabilitation (4) with very good sensitivity (4, 18).

Participants completed the 5 mWT to quantify comfortable gait speed. The test allowed for 2 m at each end for acceleration and deceleration (9-m walkway) (19). Time taken to traverse the middle 5 m was recorded with a stopwatch. We calculated the average speed from 2 trials. The 5 mWT is a reliable and valid measure of gait speed in acute and sub-acute stroke (test-retest reliability, ICC_3,1_ > 0.97) (20, 21), and is a predictor of falls in stroke survivors returning home after rehabilitation (22).

The 2 MWT was used to measure walking endurance. Participants were instructed to walk continuously along a straight corridor in the hospital unit, turning at each end, with the goal to cover as much ground as possible in 2 min. They could use their assistive devices and bracing as needed. Rests were permitted, but time was not paused. Compared to the more traditional 6-minute walk test, the 2 MWT reduces the burden of testing and minimizes potential for fatigue in the more acute recovery stage after stroke (23, 24). Indeed, the 2-minute walk test has greater reliability in inpatient stroke rehabilitation than the 6-minute walk test, although both are acceptable (24).

In the 5xSTS test, participants were timed to complete 5 repetitions of sit-to-stand transitions as quickly as possible from a standard chair without using their upper limbs. Thus, the test is considered a measure of lower extremity functional strength (25). AFO or bracing support could be worn but assistive devices were not permitted. Since several participants were unable to perform the test without the use of their upper limbs and would therefore be omitted through listwise deletion in the analysis of time to compete 5 repetitions, we calculated a repetitions per second (reps/s) variable. Using this metric, individuals who were unable to perform any repetitions without upper limb support received a score of 0. Lower extremity muscle strength measured with this test has been found to explain a significant amount of the variance in balance, falls self-efficacy, and functional mobility in people with stroke (26).

The Walk-12 (27) is a 12-item self-report scale used to measure the perceived impact of stroke on walking. We adapted the item stem from “In the last 2 weeks” to “Since your stroke.” Summed scores (range 12–60) were transformed to scores 0–100, with higher scores indicating greater self-perceived limitation in walking ability. The Walk-12 has very good psychometric properties in individuals receiving inpatient rehabilitation post stroke (27) and is related to gait performance tests in ambulatory stroke survivors (28).

The ABC is a 16-item questionnaire in which participants rate their confidence (0, no confidence to 100, complete confidence) in their ability “to maintain your balance and remain steady” in various everyday mobility situations. The average self-efficacy rating from the 16 items was calculated as the total score, with higher scores indicating higher balance self-efficacy. The ABC has been reported to be a strong predictor of future falls among older adults (29).

Cognitive-motor dual-task gait speed was assessed by having the participants walk continuously while performing a category naming task. Difficulty performing a verbal task while walking is known to be associated with falls after stroke (8, 30, 31). The details of the dual-task assessment in this study have been described elsewhere (32).

### Prospective fall tracking

After discharge, participants were followed prospectively for 3 months to monitor falls. Participants used a fall calendar provided at discharge to track falls. A fall was operationally defined as “unintentionally coming to the ground or some lower level for some reason other than as a consequence of sustaining a violent blow, loss of consciousness, or sudden onset of paralysis as in stroke or epileptic seizure” (33). A faller was defined as a person who reports ≥1 fall in the follow-up period. Participants were also contacted by telephone every 2 weeks to ask about falls. At 3-months post discharge, participants who had reported at least one fall were classified as “fallers.” There were 21 (47%) fallers and 24 (53%) non-fallers.

### Data analyses

We first compared fallers and non-fallers on biological variables (age and sex) to determine whether these needed to be controlled for in between-group comparisons of modifiable variables. Since fallers and non-fallers did not differ on age and sex (Table 2), differences between fallers and non-fallers on all modifiable factors were examined using independent sample *t*-tests for continuous normally distributed variables, Mann-Whitney *U* test for continuous non-normally distributed variables, and chi-square tests or logistic regression for categorical variables. Binocular visual acuity was classified as good (Snellen fraction in feet ≤20/25), moderately impaired (20/30 to 20/80), or poor (≥20/100) (34). Unilateral neglect was classified according to the laterality index on the Star Cancellation test. The laterality index is the ratio of stars cancelled on the left/right side of page to the total number of stars. Unilateral neglect is indicated by laterality index ≤0.46 (left unilateral neglect) or ≥0.54 (right unilateral neglect) (35).

Effect sizes for significant between-group differences were computed and are displayed in Table 2. The variables with the largest effects sizes for differentiating fallers and non-fallers were aggregated to generate a composite score. Several variations of the scoring scheme for the index were examined to try to optimize discriminative validity, as explained in the results, and the overall index accuracy was assessed by computing the AUC (C statistic) with 95% confidence intervals. The likelihood ratio test was used to compare differences between each version of the composite index and the obstacle-crossing test alone, and between index versions. Goodness of fit was assessed with the Hosmer-Lemeshow test, with *p* > 0.05 indicating good fit. Somers’ *d* is presented to indicate the agreement between the dependent variable (fall status) and independent variable (index score).

### Model validation methods

Model validation was performed using an independent sample of 30 participants with stroke enrolled in a separate study at a different hospital between January 2021 and April 2022. The participants were selected according to the same eligibility criteria with three exceptions: permitted age was 18 years and older, patients with cerebellar stroke were not excluded, and all participants were being discharged from acute inpatient rehabilitation (none directly home from acute care). Due to some limited data in the second cohort, we also examined model validation by merging the two cohorts (*n* = 75) for logistic regression with bootstrapping (1,000 samples) to generate bias-corrected 95% confidence intervals for the odds ratios.

## Results

### Participant characteristics

Twenty-one (47%) of the 45 participants had a least one fall in the first three months after discharge. Their characteristics and the circumstances of their falls have been described elsewhere (11) but the comparisons in the biologic and modifiable variables of interest in this analysis are summarized in Table 2. Non-fallers had a significantly shorter overall length of stay in hospital, which was due to 13/24 non-fallers being discharged directly home from the acute care hospital. When considering only those who received acute inpatient rehabilitation, there was no significant difference in length of stay between fallers and non-fallers. There were no differences between fallers and non-fallers in multi-morbidity, type of stroke, side of hemiplegia, or severity of depression at discharge. A larger number of non-fallers had impaired visual acuity at discharge, but the association between visual acuity and fall status was not significant (Table 2). Only one participant had unilateral neglect and only one participant had an aphasia quotient score indicative of aphasia; both were non-fallers. There was a significant difference between fallers and non-fallers on the Montreal Cognitive Assessment (Table 2), however, because the effect size was small and not clinically important, the Montreal Cognitive Assessment was not considered for inclusion in the discharge fall-risk composite index.

### Index model development

The dependent variable was fall status at 3 months post discharge from hospital to home. Table 2 illustrates that fallers and non-fallers differed at discharge on all the physical performance and self-reported measures. There was also an association between fall status and type of assistive device, but not use of an AFO/ankle brace. Individuals who used a single-point cane (*n* = 8, 18%) or a quad cane (*n* = 9, 20%) at discharge had significantly higher odds of being a faller than a person who did not use an assistive device (*n* = 17, 38%), with odds ratios of 9.8 (95% CI, 1.4–68.8) and 11.4 (95% CI, 1.7–78.4), respectively. Participants who used a walker at discharge (*n* = 11, 24%) were also more likely to be a faller than a person who did not us an assistive device, but the association was not significant (odds ratio 1.9, 95% CI, 0.4–9.8).

The ABC score and 5xSTS had the weakest effect sizes (Cohen’s *d *≤ 0.66; Table 2). Thus, in the interests of developing the strongest and most concise composite measure, the ABC and 5xSTS were excluded from further consideration. Dual-task gait speed also had only a moderate effect size and was discarded for aggregation. None of the participants required a rest break during the 2 MWT, but two participants (both in an inpatient rehabilitation facility as opposed to acute care hospital) were unable to complete the 2 MWT due to fatigue. This raised some concern about how feasible the 2 MWT would be to administer routinely in acute inpatient rehabilitation. Taken together with the observation that the 2 MWT was no stronger in differentiating fallers and non-fallers than the 5 mWT (both Cohen’s *d *= 0.86), we excluded the 2 MWT from further consideration. The paretic limb step test score was stronger than the non-paretic limb score for differentiating fallers and non-fallers, so we considered only the paretic limb score further. Therefore, 5 predictors were considered for index model derivation: the obstacle-crossing test, paretic-limb step test, and 5 mWT, Walk-12, and assistive device type. With a sample size of 45, we had *n* = 9 per predictor, which approximates the widely accepted 10 events per variable for binary logistic regression (36). Moreover, we were only entering a single composite predictor into the model each time, thus the sample size was considered acceptable for this proof-of-concept demonstration.

**Figure 1:**
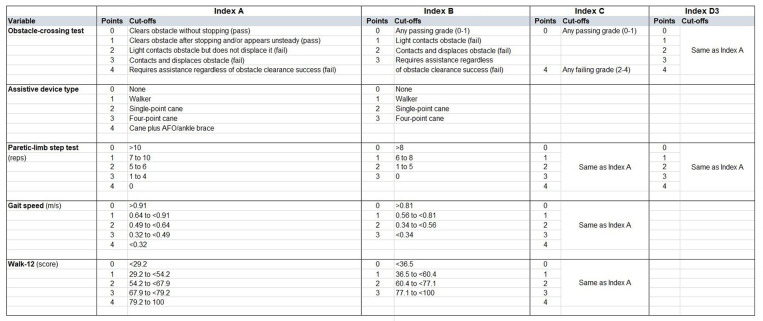
Composite index variations with cut-offs for each item.

We first constructed an index comprising all 5 variables (Index A). We created a 5-point (0–4) scale for each variable. For the obstacle-crossing test, we used the 5-point grade score as defined in the Methods. For assistive device use, we assigned 0 points to no assistive device, then 1, 2, or 3 points for walker, single-point cane, and quad cane, respectively, which was based on rank order of beta coefficient from the logistic regression. To create the fifth category, we assigned 4 points for individuals using a cane (either type) with an AFO/brace. We discretized the 3 continuous variables (step test, 5 mWT, Walk-12) into 5 categories using quintiles as cut offs (Figure 1). Thus, the total score for Index A ranged from 0 to 20 with higher scores indicating higher fall risk.

We then created an alternative index (Index B) using the same variables as Index A but modifying the 5-point scale to a 4-point scale (Figure 1). For Index B, the two obstacle-test “pass” scores (i.e., 0 or 1) were collapsed, since only 3/25 passes scored 1. The “fail” grades of 2, 3, 4 were assigned 1, 2, 3 points, respectively. For assistive device use, we omitted the category of “cane plus AFO” and retained the 0–3 scoring scheme as Index A. For the continuous variables, quartiles were used to generate cut offs. Thus, the total score for Index B ranged from 0 to 15 with higher scores indicating higher fall risk.

In a third version (Index C), we omitted assistive device type from Index A, since we had previously observed that assistive device use was a potential factor contributing to misclassification of fall status on the obstacle test (11). To further simplify, we also binarized the obstacle test as 0 (any passing grade) or 4 points (any failing grade), since 80% of all scores were 0 or 4. Thus, the total score for Index C ranged from 0 to 16 with higher scores indicating higher fall risk.

Finally, given our previous observation that use of an assistive device to perform the obstacle test was likely contributing to the high false negative rate (11), we created a simplified index (Index D) aggregating only the obstacle test and the step test (which is performed without assistive devices or upper limb support). We examined this combination in 3 ways. First, we used binarized scores for the obstacle (0 pass, 1 fail) and binarized scores for the paretic-limb step test (<7 fail, ≥7 pass) (4) and assigned 0 (pass) to participants who passed both tests and 1 (fail) to participants who failed either or both tests. Thus, this version of Index D was a binary outcome of overall pass or fail (Index D1). Second, we summed the binarized scores for each test such that 0 represented pass on both tests, 1 represented a fail on one test, and 2 represented a fail on both tests. Thus, this version of Index D was 3-level categorical outcome (Index D2). Third, we summed the 0–4-point scores for both the obstacle and step tests from Index A for a total score of 0–8 (Index D3) (Figure 1).

### Model performance

Index scores for each index version were entered as the single predictor in a binary logistic regression to estimate the probability of being a faller (with 95% confidence intervals). Differences between any two models were examined by nesting one model within another and using a likelihood ratio test for comparison. Discrimination was examined by calculating the AUC (C statistic) with 95% confidence intervals. Sensitivity, specificity, and positive and negative likelihood ratios and their respective 95% confidence intervals were also calculated for each index version (Table 3).

**Table 3:** Metrics for each index version and the obstacle-crossing test from the model derivation sample (*n* = 45) for predicting fall status at 3-months post discharge. Cut off scores were determined from ROC analysis. Values above the cut off indicate high fall risk. Somer's d is for the binary variable using the indicated cut-off scores.

Test	Cut off	Sensitivity (95% CI)	Specificity (95% CI)	LR+ (95% CI)	LR– (95% CI)	AUC (95% CI)	Somers’ *d*
Obstacle-crossing test	Pass/fail	0.67 (0.43–0.85)	0.83 (0.63–0.95)	4.00 (1.56–10.28)	0.40 (0.21–0.75)	0.75 (0.60–0.90)	0.519, *p* < .001
Index A (0–20 points)	≥9.5	0.71 (0.48–0.89)	0.75 (0.53–0.90)	2.86 (1.36–6.01)	0.38 (0.19–0.78)	0.83 (0.71–0.95)	0.464, *p* < .001
Index B (0–15 points)	≥5.5	0.90 (0.70–0.99)	0.67 (0.45–0.84)	2.71 (1.52–4.86)	0.14 (0.04–0.55)	0.82 (0.70–0.95)	0.593, *p* < .001
Index C (0–16 points)	≥6.5	0.86 (0.64–0.97)	0.67 (0.45–0.84)	2.57 (1.42–4.65)	0.21 (0.07–0.63)	0.85 (0.74–0.96)	0.534, *p* < .001
Index D3 (0–8 points)	≥3.5	0.71 (0.48–0.89)	0.83 (0.63–0.95)	4.29 (1.68–10.91)	0.34 (0.17–0.69)	0.85 (0.73–0.96)	0.559, *p* < .001

AUC denotes area under the ROC curve, LR+/LR– denotes positive/negative likelihood ratio.

Higher LR+ values are better, lower LR– values are better.

Of the 3 variations of Index D, Index D3 had superior goodness of fit compared to Index D1 [*χ*^2^(1) = 5.227, *p* = 0.022] and Index D2 [*χ*^2^(2) = 11.250, *p* = 0.004]. Thus, Index D1 and D2 were not examined further. All versions of the composite index (A, B, C, D3) were significantly better than the obstacle test alone, *χ*^2^(1) ≥ 4.259, *p* < 0.04 or better. There were no significant differences between any of the 4 versions of the index, but Index C had the best fit of all versions. The performance of each model is summarized in Table 3.

For Index C, the best composite test, which had a score ranging from 0 to 16, ROC analysis indicated that sensitivity (86%) and specificity (67%) were optimized at a cut-off score ≥6.5 points, with ≥6.5 points indicating fall risk. The specificity of Index C was impacted by 8 false positives. That is, one third of non-fallers had “high fall risk” on Index C, 4 of these had failed the obstacle test. Further inspection of the false positive cases indicated that these non-fallers had significantly poorer performance on the obstacle-crossing test, paretic-limb step test, 5 mWT, and Walk 12 than the other non-fallers (true negatives). False positives on Index C were also significantly more likely than the true negatives to be using an assistive device at discharge. Thus, there appears to be a subgroup of low-functioning, high fall risk individuals who do not fall after discharge.

Although the goodness of fit of Index D3 was not significantly different than Index C, there was less asymmetry between the sensitivity (71%) and specificity (83%) than for Index C (86% and 67%, respectively).

### Model validation

A separate sample of patients from Spaulding Rehabilitation Hospital (Boston, MA) who were participating in a study that focused on examining variations of the obstacle-crossing test were used to validate the models (Index C and Index D3 only). All participants had completed the obstacle-crossing test identical to the test in the derivation study. Of the 45 enrolled participants in the study Spaulding Rehabilitation Hospital, 30 had currently completed 3-month follow-up and comprised the external validation sample. The Walk-12 was not included in the original research protocol at Spaulding Rehabilitation Hospital, so we had too few new participants with Walk-12 discharge scores and 3-month fall status to validate Index C in the new sample alone. Thus, Index C was validated by adding *n* = 9 new participants to the derivation study cohort (total *n* = 54) and testing the stability of the model in 1,000 bootstrap samples. Index D3 was validated in the external sample of *n* = 30, and the stability of the model was examined in 1,000 bootstrap samples replaced from the pooled cohort of *n* = 75. The performance of Index C and Index D3 from the validation analysis is presented in Table 4.

**Table 4:** Metrics for Index C and Index D3 from the model validation samples for predicting fall status at 3-months post discharge. Cut off scores established from the model derivation were applied to the validation samples. Values above the cut off indicate high fall risk. Somer's d is for the binary variable using the indicated cut-off scores.

Test	*N*	Cut off	Sensitivity (95% CI)	Specificity (95% CI)	LR+ (95% CI)	LR– (95% CI)	AUC (95% CI)	Somers’ *d*
Index C (0–16 points)	54	≥6.5	0.77 (0.56–0.91)	0.68 (0.48–0.84)	2.39 (1.34–4.27)	0.34 (0.16–0.72)	0.82 (0.71–0.93)	0.450, *p* < .001
Index D3 (0–8 points)	30	≥3.5	0.56 (0.21–0.86)	0.95 (0.76–1.00)	11.67 (1.58–86.21)	0.47 (0.22–0.97)	0.84 (0.68–1.00)	0.667, *p* = .008

AUC denotes area under the ROC curve, LR+/LR– denotes positive/negative likelihood ratio.

Higher LR+ values are better, lower LR– values are better.

In the validation sample of *n* = 54 (*n* = 26 fallers, *n* = 28 non-fallers), Index C was a significant predictor of fall status, OR 1.3, with the bias-corrected 95% confidence interval for the odds ratio generated from bootstrapping estimated to be 1.1–1.7 (*p* < 0.001). The index had excellent discrimination ability with AUC 0.82 (95% CI, 0.71–0.93). Using the previously established cut-off from the model derivation sample (≥6.5 points), Index C had good sensitivity and fair specificity (Table 4).

In the external sample of *n* = 30 (*n* = 9 fallers, *n* = 21 non-fallers), Index D3 had acceptable fit (Hosmer and Lemeshow test *p* = 0.588) and significantly predicted fall status, odds ratio 2.0 (95% CI, 1.2–3.4). The bias-corrected 95% confidence interval for the odds ratio for Index D3 generated from bootstrapping the pooled cohort (*n* = 75) was 1.4–2.6 (*p* < 0.001). The index had excellent discrimination ability with AUC 0.84 (95% CI, 0.68–1.00). However, using the previously established cut-off from the model derivation sample (≥3.5 points), Index D3 had poor sensitivity and excellent specificity (Table 4). The ROC analysis on the external validation sample (*n* = 30), found that sensitivity (0.67, 95% CI, .30–0.93) and specificity (0.90, 95% CI, 0.70–0.99) were optimized with a cut off of ≥2.5 points. Thus, further validation in larger samples is needed to confirm optimal cut off score for this Index.

## Discussion

The purpose of this study was to demonstrate proof-of-concept that aggregating a limited number of highly discriminatory measures with the obstacle-crossing test would at least partially compensate for its limitations in sensitivity. Indeed, we found that by strategically combining the discharge variables that had the strongest effect sizes for differentiating fallers and non-fallers, we could generate a concise, composite index that significantly improved prediction accuracy compared to the obstacle test alone (AUC 0.74 vs. AUC 0.85, see Table 3). Furthermore, the sensitivity of the best version of index (Index C, 86%) was substantially greater than the obstacle test alone (67%). The improvement in sensitivity was likely due to the aggregation of the obstacle test with the step test because the step test is a similar dynamic balance activity but without the advantage of upper extremity support from unilateral or bilateral assistive devices. This was corroborated by the analysis of Index D3, which showed that summing the discretized scores for only the obstacle and step test was not significantly different than the predictive accuracy of the 4-item Index C. However, Index C had better sensitivity (83%) than the obstacle-step test combination in Index D3 (71%) and the obstacle test alone (67%). Thus, the 4-item index was slightly better at correctly classifying fallers.

The improved sensitivity in Index C, however, came at a cost to specificity (ability to classify non-fallers). Whereas the obstacle test had few false positives (*n* = 4, specificity 83%), Index C had twice as many false positives (*n* = 8, specificity 67%). One could argue that lower specificity is not as clinically worrisome as low sensitivity. With low sensitivity, a large number of fallers are missed, which could have important safety implications. With low specificity, many individuals who are predicted to be fallers do not ultimately fall. Our analyses revealed that this subgroup of non-fallers (false positives) is quite likely at high risk of falling, based on their poorer physical performance and self-rated walking ability, so there must be some other reason they are not falling. One plausible explanation could be cautious or avoidant behavior due to fear of falling that may minimize opportunity to fall. A further possible explanation is that these individuals may have low levels of physical activity, either due to a habitual sedentary lifestyle or limited post-discharge physical activity due to functional limitations or inadequate social support. It is known that people who fall after stroke have higher activity levels than non-fallers (37). Yet another possible explanation is that these individuals received ongoing rehabilitation after discharge, which may have improved their physical functioning and reduced their risk of falling. Finally, the 3-month follow up period may have been too short to reveal a fall in some individuals at risk of falling. Considering these possible explanations, this type of misclassification by the test/index, may not necessarily be a fault of the test. However, future research should examine if the predictive validity of fall risk assessed at discharge is moderated by rehabilitation after hospital discharge, altered by duration of follow-up period to classify fallers and non-fallers, or influenced by habitual (pre-stroke) or post-stroke physical activity.

All 4 versions of our composite index demonstrated better predictive accuracy than what is currently estimated for existing clinical measures in inpatient rehabilitation. For example, in a previous study, the sensitivity and specificity of the BBS for predicting future fallers from inpatient rehabilitation discharge was only 63% and 65%, respectively (cut-off score <45, *n* = 141) (2). BBS accuracy (cut-off score of 46.5 points, *n* = 50) was slightly better in chronic stroke survivors when predicting fallers and non-fallers in the last 12 months: 75% sensitivity, 77% specificity, and AUC 81% (38). Yet, in another study, BBS (cut-off score of 52 points, *n* = 50) had excellent sensitivity (91%) but uninformative specificity (42%), with AUC 72%, for classifying chronic stroke survivors with and without a history of falls (26). Our brief composite measures are consistently better than these reports for the BBS.

Similarly, the Timed Up and Go test (TUG) has been associated with history of falls in chronic stroke (7, 39), but when used in inpatient rehabilitation to prospectively predict post-stroke fallers at 6 or 12 months post discharge, TUG ≥14 s had poor sensitivity (50%) but good specificity (78%, *n* = 105) (2). Ng and colleagues (22) found that the TUG was not associated with falls in people with stroke being discharged from rehabilitation, but they included a large number of participants who had a pre-stroke history of falling, including 12% of non-fallers (identified prospectively) with a history of falling in the 12 months prior to stroke. Sahin et al. (26) also found that the TUG could not differentiate stroke survivors with and without a history of falls (AUC 0.42, 95% CI, 0.29–0.64), but these were chronic post-stroke individuals who were mostly unlimited community ambulators. There is currently very little quality evidence that can guide selection of clinical instruments for accurate prediction of future post-stroke fallers during inpatient rehabilitation. This study provides compelling, validated proof-of-concept that simple aggregation of ecologically valid mobility tasks (obstacle negotiation, step test) with or without more traditional measures (gait speed, self-rated walking disability, assistive device use) may yield highly accurate prediction of probability of falling in the first 3 months after discharge.

The Functional Gait Assessment (FGA) and the Dynamic Gait Index (DGI)/modified DGI (mDGI) are existing multi-item clinical assessments used to assess fall risk that include stepping over an obstacle (one or two stacked shoeboxes). Although the FGA and DGI are commonly used in inpatient rehabilitation and have established reliability and validity in stroke (40), there have not yet been any studies that have examined their predictive accuracy for classifying future fallers and non-fallers at inpatient discharge. Indeed, there are no existing estimates of the predictive validity of the FGA in stroke (subacute or chronic). In community-dwelling older adults without stroke, the FGA (≤22/30) and the DGI (≤20/24) had excellent accuracy to classify fall risk (AUC 0.92 and 0.91, respectively) (41). In a mixed neurological sample, of which 26% were adults with chronic stroke, at the optimal cut-off score, the mDGI had 50% sensitivity and 100% sensitivity for 68% overall accuracy (95% CI, 0.43–0.93) for differentiating fallers and non-fallers. The FGA and DGI/mDGI should be examined for their accuracy to classify fall risk in subacute stroke/inpatient rehabilitation. However, a potential limitation of the FGA and DGI is that they include exclusively walking items and allow assistive device use. Therefore, they risk being prone to the same shortfalls we have observed in our obstacle test.

An important clinical implication worth noting is the relative brief amount of time and equipment required to administer the combination of tests in Index C and Index D3. Our previous research found that only two trials of the obstacle crossing test are probably needed (11), so the obstacle-crossing test can be set up and completed in about 5 min. The step test is a maximum of 15 s per lower limb. Two trials of the 5 mWT take 1–2 min or less, and the Walk12 can be administered, in our experience, in fewer than 5 min. Thus, Index C and Index D3 can be administered clinically in 10–12 min or 6 min, respectively. By comparison, the BBS takes approximately 15–20 min to administer by an experienced clinician (42). The FGA is not as lengthy as the BBS and is estimated to take about 10 min to administer by an experienced clinician (42). However, as discussed earlier, its validity for predicting future fallers prior to discharge from inpatient rehabilitation is unknown. We are not proposing that the BBS and FGA should be discontinued in clinical practice. Indeed, these multi-item assessments provide clinicians with useful information about specific balance and mobility limitations that can guide treatment. However, current evidence indicates that these clinical instruments are limited in their ability to accurately identify future post-stroke fallers. A brief composite mobility index, such as those derived in this proof-of-concept study, may provide a quick, accurate, and reliable assessment of fall risk that can be administered periodically during inpatient rehabilitation without consuming valuable therapy time.

There are several limitations of this study that must be considered. Our physical performance battery, which provided the source of predictor variables for this proof-of-concept demonstration, did not include any measures of reactive balance (e.g., lean-and-release postural responses) or sensory factors (e.g., protective sensation on the plantar surface of the feet, proprioception), or the number of medications and use of centrally acting medications, both of which are related to fall risk (22, 26). Although we assessed visual acuity, visual acuity may not be the most important visual risk factor for falls (43). Rather, depth perception and contrast sensitivity, both of which can be affected in the inferior visual field when wearing multifocal glasses, appear to be the most important visual factors contributing to fall risk, at least in older adults (43). We excluded individuals with cerebellar stroke, but that may need to be reconsidered to improve external validity. That said, the models were externally validated in a sample that included 7 participants with cerebellar stroke (*n* = 3 fallers, *n* = 4 non-fallers). Further validation in larger samples is needed to determine optimal cut offs and model accuracy. However, this study was intended to demonstrate proof-of-concept and further model development may need to be considered before validating these models further. Although the 3-month follow up period in this study was relatively short, and high fall rates after stroke are reported up to 6 months post discharge, as many as 28% of patients fall in the first 2 weeks of going home (44) and 52% (11) to 58% (5) of first falls occur in the first month after discharge. Indeed, the first two months after discharge have been identified as a critical time point for falls (5, 45). Thus, it may be most important to identify the patients who are likely to fall in the first 3 months of going home so that these high-risk individuals can be prioritized for post-discharge rehabilitation immediately. Unfortunately, we do not have detailed data on whether falls occurred in the context of greater amounts of physical activity. It is known that people who fall after stroke have higher reported activity levels than non-fallers (37). Accordingly, monitoring physical activity and fear of falling avoidance behavior after discharge may be an important component of fall risk assessment validation and to understand why some “high risk” individuals do not fall (i.e., false positives). Conversely, it would be helpful to know if fallers who had not been predicted to fall (i.e., false negatives) fell because they were engaging in relatively high-risk activities. Although we found that most falls occurred in the home, as reported elsewhere (11), we did observe that false negatives on the obstacle test were more likely to report falling outdoors during vigorous activity, such as while performing yard work or hiking. Fall typology needs more rigorous evaluation in a larger sample to explore whether predictive validity of individual tests or test combinations varies for different faller types. Finally, we binarized and/or discretized the predictors for ease of scoring. It is possible that retaining the continuous structure of variables could improve model fit, however, we believe that to facilitate implementation and regular adoption in clinical practice, a fall-risk composite index should have a simple scoring scheme that is pragmatic for bedside administration and interpretation.

## Conclusion

This study provides convincing proof-of-concept that strategic aggregation of performance-based and self-reported mobility measures, including a novel and demanding obstacle-crossing test, can predict future post-stroke fallers at discharge with excellent accuracy. The findings also suggest that clinical assessment of fall risk in inpatient rehabilitation most likely needs to include at least one dynamic balance task that does not allow use of upper limb support or assistive devices, as this advantage may inflate false negatives and impede clinical decision making. Existing clinical instruments that include more ecologically complex mobility tasks, such as stepping over an obstacle, require validation of predictive accuracy in the inpatient stroke rehabilitation setting. Further development of a simple composite fall-risk index for inpatient rehabilitation is needed, given the limitations of existing outcome measures and the lack of knowledge of longitudinal association to predict future post-stroke fallers among inpatient stroke survivors who have not yet fallen.

## Data Availability

The raw data supporting the conclusions of this article will be made available by the authors, without undue reservation.

## References

[1] Nyberg L Gustafson Y. Patient falls in stroke rehabilitation. A challenge to rehabilitation strategies. Stroke. (1995) 26(5):838–42. 10.1161/01.STR.26.5.838 7740577

[2] Andersson AG Kamwendo K Seiger A Appelros P. How to identify potential fallers in a stroke unit: validity indexes of 4 test methods. J Rehabil Med. (2006) 38(3):186–91. 10.1080/16501970500478023 16702086

[3] Forster A Young J. Incidence and consequences of falls due to stroke: a systematic inquiry. Br Med J. (1995) 311:83–6. 10.1136/bmj.311.6997.83 7613406 PMC2550147

[4] Mackintosh SF Hill KD Dodd KJ Goldie PA Culham EG. Balance score and a history of falls in hospital predict recurrent falls in the 6 months following stroke rehabilitation. Arch Phys Med Rehabil. (2006) 87(12):1583–9. 10.1016/j.apmr.2006.09.004 17141637

[5] Mackintosh S Hill K Dodd K Goldie P Culham E. Falls and injury prevention should be part of every stroke rehabilitation plan. Clin Rehabil. (2005) 19(4):441–51. 10.1191/0269215505cr796oa 15929514

[6] Mansfield A Wong JS McIlroy WE Biasin L Brunton K Bayley M Do measures of reactive balance control predict falls in people with stroke returning to the community? Physiotherapy. (2015) 101(4):373–80. 10.1016/j.physio.2015.01.009 26050134

[7] Pinto EB Nascimento C Monteiro M Castro M Maso I Campos A Proposal for a new predictive scale for recurrent risk of fall in a cohort of community-dwelling patients with stroke. J Stroke Cerebrovasc Dis. (2016) 25(11):2619–26. 10.1016/j.jstrokecerebrovasdis.2016.06.045 27475520

[8] Hyndman D Ashburn A. People with stroke living in the community: attention deficits, balance, ADL ability and falls. Disabil Rehabil. (2003) 25(15):817–22. 10.1080/0963828031000122221 12851091

[9] Taylor-Piliae RE Hoke TM Hepworth JT Latt LD Najafi B Coull BM. Effect of Tai Chi on physical function, fall rates and quality of life among older stroke survivors. Arch Phys Med Rehabil. (2014) 95(5):816–24. 10.1016/j.apmr.2014.01.001 24440643

[10] Said CM Galea MP Lythgo N. People with stroke who fail an obstacle crossing task have a higher incidence of falls and utilize different gait patterns compared with people who pass the task. Phys Ther. (2013) 93(3):334–44. 10.2522/ptj.20120200 23064734

[11] Feld JA Goode AP Mercer VS Plummer P. Utility of an obstacle-crossing test to classify future fallers and non-fallers at hospital discharge after stroke: a pilot study. Gait Posture. (2022) 96:179–84. 10.1016/j.gaitpost.2022.05.037 35667230 PMC9535661

[12] Gitlin LN Schemm RL Landsberg L Burgh D. Factors predicting assistive device use in the home by older people following rehabilitation. J Aging Health. (1996) 8(4):554–75. 10.1177/089826439600800405 10182386

[13] Venti M. Cerebellar infarcts and hemorrhages. Front Neurol Neurosci. (2012) 30:171–5. 10.1159/000333635 22377889

[14] Sarikaya H Steinlin M. Cerebellar stroke in adults and children. Handb Clin Neurol. (2018) 155:301–12. 10.1016/B978-0-444-64189-2.00020-2 29891068

[15] Perry J Garrett M Gronley JK Mulroy SJ. Classification of walking handicap in the stroke population. Stroke. (1995) 26(6):982–9. 10.1161/01.STR.26.6.982 7762050

[16] Patla AE Prentice SD Robinson C Neufeld J. Visual control of locomotion: strategies for changing direction and for going over obstacles. J Exp Psychol Hum Percept Perform. (1991) 17(3):603–34. 10.1037/0096-1523.17.3.603 1834781

[17] Hill K Bernhardt J McGann A Maltese D Berkovits D. A new test of dynamic standing balance for stroke patients: reliability, validity and comparison with healthy elderly. Physiother Can. (1996) 48(4):257–62. 10.3138/ptc.48.4.257

[18] Blennerhassett J Dite W Ramage E Richmond M. Changes in balance and walking from stroke rehabilitation to the community: a follow-up observational study. Arch Phys Med Rehabil. (2012) 93(10):1782–7. 10.1016/j.apmr.2012.04.005 22522218

[19] Collen FM Wade DT Bradshaw CM. Mobility after stroke: reliability of measures of impairment and disability. Int Disabil Stud. (1990) 12(1):6–9. 10.3109/03790799009166594 2211468

[20] Cunha IT Lim PA Henson H Monga T Qureshy H Protas EJ. Performance-based gait tests for acute stroke patients. Am J Phys Med Rehabil. (2002) 81(11):848–56. 10.1097/00002060-200211000-00008 12394997

[21] da Cunha-Filho IT Henson H Wankadia S Protas EJ. Reliability of measures of gait performance and oxygen consumption with stroke survivors. J Rehabil Res Dev. (2003) 40(1):19–25. 10.1682/JRRD.2003.01.0019 15150717

[22] Ng MM Hill KD Batchelor F Burton E. Factors predicting falls and mobility outcomes in patients with stroke returning home after rehabilitation who are at risk of falling. Arch Phys Med Rehabil. (2017) 98(12):2433–41. 10.1016/j.apmr.2017.05.018 28647551

[23] Billinger SA Arena R Bernhardt J Eng JJ Franklin BA Johnson CM Physical activity and exercise recommendations for stroke survivors: a statement for healthcare professionals from the American Heart Association/American Stroke Association. Stroke. (2014) 45(8):2532–53. 10.1161/STR.0000000000000022 24846875

[24] Kosak M Smith T. Comparison of the 2-, 6-, and 12-minute walk tests in patients with stroke. J Rehabil Res Dev. (2005) 42(1):103–7. 10.1682/JRRD.2003.11.0171 15742254

[25] Csuka M McCarty DJ. Simple method for measurement of lower extremity muscle strength. Am J Med. (1985) 78(1):77–81. 10.1016/0002-9343(85)90465-6 3966492

[26] Belgen B Beninato M Sullivan PE Narielwalla K. The association of balance capacity and falls self-efficacy with history of falling in community-dwelling people with chronic stroke. Arch Phys Med Rehabil. (2006) 87(4):554–61. 10.1016/j.apmr.2005.12.027 16571397

[27] Holland A O’Connor RJ Thompson AJ Playford ED Hobart JC. Talking the talk on walking the walk: a 12-item generic walking scale suitable for neurological conditions? J Neurol. (2006) 253(12):1594–602. 10.1007/s00415-006-0272-2 16924398

[28] Brogårdh C Flansbjer UB Lexell J. Self-reported walking ability in persons with chronic stroke and the relationship with gait performance tests. PM / R. (2012) 4(10):734–8. 10.1016/j.pmrj.2012.05.004 22766045

[29] Landers MR Oscar S Sasaoka J Vaughn K. Balance confidence and fear of falling avoidance behavior are most predictive of falling in older adults: prospective analysis. Phys Ther. (2016) 96(4):433–42. 10.2522/ptj.20150184 26294679

[30] Baetens T De Kegel A Palmans T Oostra K Vanderstraeten G Cambier D. Gait analysis with cognitive-motor dual tasks to distinguish fallers from nonfallers among rehabilitating stroke patients. Arch Phys Med Rehabil. (2013) 94(4):680–6. 10.1016/j.apmr.2012.11.023 23187040

[31] Hyndman D Ashburn A. “Stops walking when talking” as a predictor of falls in people with stroke living in the community. J Neurol Neurosurg Psychiatr. (2004) 75(7):994–7. 10.1136/jnnp.2003.016014 PMC1739145 15201358

[32] Feld JA Plummer P. Patterns of cognitive-motor dual-task interference post stroke: an observational inpatient study at hospital discharge. Eur J Phys Rehabil Med. (2021) 57(3):327–36. 10.23736/S1973-9087.20.06273-5x 32935952

[33] Gibson M. Falls in later life. Improving the health of older people. New York: Oxford University Press (1990).

[34] Felson DT Anderson JJ Hannan MT Milton RC Wilson PW Kiel DP. Impaired vision and hip fracture. The framingham study. J Am Geriatr Soc. (1989) 37(6):495–500. 10.1111/j.1532-5415.1989.tb05678.x 2715555

[35] Zelter L Menon A. Star Cancellation Test: Stroke Engine (2022) [cited 2022 26 Jun 2022]. Available from: https://strokengine.ca/en/assessments/star-cancellation-test/

[36] Moons KG de Groot JA Bouwmeester W Vergouwe Y Mallett S Altman DG Critical appraisal and data extraction for systematic reviews of prediction modelling studies: the CHARMS checklist. PLoS Med. (2014) 11(10):e1001744. 10.1371/journal.pmed.1001744 25314315 PMC4196729

[37] Kerse N Parag V Feigin VL McNaughton H Hackett ML Bennett DA Falls after stroke: results from the Auckland Regional Community Stroke (ARCOS) Study, 2002 to 2003. Stroke. (2008) 39(6):1890–3. 10.1161/STROKEAHA.107.509885 18483413

[38] Sahin IE Guclu-Gunduz A Yazici G Ozkul C Volkan-Yazici M Nazliel B The sensitivity and specificity of the balance evaluation systems test-BESTest in determining risk of fall in stroke patients. NeuroRehabilitation. (2019) 44(1):67–77. 10.3233/NRE-182558 30814369

[39] Pinto EB Nascimento C Marinho C Oliveira I Monteiro M Castro M Risk factors associated with falls in adult patients after stroke living in the community: baseline data from a stroke cohort in Brazil. Top Stroke Rehabil. (2014) 21(3):220–7. 10.1310/tsr2103-220 24985389

[40] Lin JH Hsu MJ Hsu HW Wu HC Hsieh CL. Psychometric comparisons of 3 functional ambulation measures for patients with stroke. Stroke. (2010) 41(9):2021–5. 10.1161/STROKEAHA.110.589739 20671244

[41] Wrisley DM Kumar NA. Functional gait assessment: concurrent, discriminative, and predictive validity in community-dwelling older adults. Phys Ther. (2010) 90(5):761–73. 10.2522/ptj.20090069 20360052

[42] Shirley Ryan Ability Lab. Rehabilitation Measures Database Chicago, IL (2022) [cited 2022 9 Sep 2022]. Available from: https://www.sralab.org/rehabilitation-measures

[43] Lord SR. Visual risk factors for falls in older people. Age Ageing. (2006) 35(Suppl 2):ii42–5. 10.1093/ageing/afl085 16926203

[44] Batchelor FA Hill KD Mackintosh SF Said CM Whitehead CH. Effects of a multifactorial falls prevention program for people with stroke returning home after rehabilitation: a randomized controlled trial. Arch Phys Med Rehabil. (2012) 93(9):1648–55. 10.1016/j.apmr.2012.03.031 22503739

[45] Batchelor FA Mackintosh SF Said CM Hill KD. Falls after stroke. Int J. (2012) 7(6):482–90. 10.1111/j.1747-4949.2012.00796.x 22494388

